# Heterogeneous Indicators of Cognitive Performance and Performance Variability Across the Lifespan

**DOI:** 10.3389/fnagi.2020.00062

**Published:** 2020-03-06

**Authors:** Lauren A. Rutter, Ipsit V. Vahia, Brent P. Forester, Kerry J. Ressler, Laura Germine

**Affiliations:** ^1^Department of Psychological and Brain Sciences, Indiana University, Bloomington, IN, United States; ^2^Institute for Technology in Psychiatry, McLean Hospital, Belmont, MA, United States; ^3^Department of Psychiatry, Harvard Medical School, Boston, MA, United States; ^4^Division of Geriatric Psychiatry, McLean Hospital, Belmont, MA, United States; ^5^Division of Depression and Anxiety Disorders, McLean Hospital, Belmont, MA, United States

**Keywords:** variability, cognitive performance, lifespan, web, digital neuropsychology

## Abstract

Reaction time (RT) and RT variability are core components of cognitive performance that can be captured through brief and easy-to-administer tasks of simple RT and choice RT. The current study aims to describe age-related differences in cognitive performance, toward better characterizing normative performance across the lifespan. We examined mean and variability of response times on a simple RT and choice RT tasks in a large and diverse web-based sample (10,060 visitors to TestMyBrain.org). We also examined lifespan-related differences in response time variability using multiple different approaches (raw variability, mean scaled variability, and mean residualized variability). These analyses revealed significant heterogeneity in the patterns of age-related differences in performance, across metrics and within different estimates of the same metric. Based on segmented regression analysis, age of peak performance differed significantly across metrics, with young adults having the best performance based on measures of median RT, middle age adults at peak on certain measures of RT variability (standard deviation and coefficient of variability), and older adults showing the best performance based on accuracy and mean-corrected RT variability. Our results indicate that no single measure of cognitive performance and performance variability produces the same findings with respect to age related change, with further work needed to establish the validity of particular metrics for different applications.

## Introduction

Performance variability has been linked with cognitive decline (e.g., Hultsch et al., 2000, 2008; Williams et al., 2005; Haynes et al., 2018). However, given the normal reductions in cognitive performance that occur with aging, it is unknown how much different aspects of cognitive performance *should* vary within an individual, and to what degree impairments are pathological. Cognitive performance on speeded, reaction-time-based tasks can be captured in three ways: first, in terms of accuracy for measures that include correct/incorrect response options; second, in terms of mean or median reaction time (RT); and third, in terms of RT variability (e.g., West et al., 2002; Bunce et al., 2004; Deary and Der, 2005; Williams et al., 2005; Der and Deary, 2006; Gooch et al., 2009). Although the vast majority of the literature in neuropsychiatry focuses on accuracy or mean RT, here we compare measures of accuracy and mean RT to measures of RT variability to see to what degree age-related differences in each of these metrics show distinct or convergent patterns.

Variability in cognition may be a particularly important metric, in that individuals with lower baseline performance levels may be able to normally compensate across cognitive domains, whereas when their performance becomes less stable (more variable), such compensatory processes may fail (e.g., MacDonald et al., 2003; Bielak et al., 2010). For decades, researchers have posited that RT variability is an important component of intelligence (Jensen, 1992, 1993; Schmiedek et al., 2007, 2009, 2013). RT variability has been found to be sensitive to the cognitive changes that occur in psychiatric and neurological disorders, normal development, and aging (MacDonald et al., 2006, 2009; Esterman et al., 2013).

Lower RT variability (more consistent performance) has been associated with better cognitive control (Vasquez et al., 2017) and higher RT variability (less consistent performance) has been associated with cognitive instability (Fjell et al., 2011), greater mental noise (Ode et al., 2011), and poorer cognitive control (Strauss et al., 2007; Papenberg et al., 2011; Vaughan et al., 2013). Previous studies have found that RT variability follows a u-shaped curve across development and aging (see Williams et al., 2005), with variability being highest in childhood and older adulthood. While most studies on RT variability within an individual use cross-sectional data, longitudinal studies of aging show that RT variability continues to increase linearly from early adulthood into late adulthood (MacDonald et al., 2003; Lövdén et al., 2007). Typically, variability within an individual is greater when the task requires response selection or cognitive control, as opposed to tasks that require minimal cognitive control, such as a simple RT task (Bielak et al., 2010).

There have been inconsistencies in the literature, however, in terms of how RT variability differs across the lifespan. Different measures of RT variability can produce different results (McAuley et al., 2006; Dykiert et al., 2012). One of the sources of divergence in results is the degree to which measures of RT variable are adjusted for differences in mean RT. Since RT variability (computed in terms of the standard deviation in RTs) tends to increase with mean RT, differences in RT variability could reflect differences in mean RT. In a systematic review and meta-analysis, Dykiert et al. (2012) found that effect sizes were larger for variability measures that did not adjust for differences in mean RT than those that did, with some studies that used mean-adjusted RT variability metrics showing little to no increase in RT variability in older age. Here, we focus on variability in RTs within a task, rather than variability across trials within the same session (dispersion) or variability in scores at different timepoints (e.g., longitudinal variability, measurement burst designs, or ecological momentary assessment). While the latter forms of variability are potentially important and informative (e.g., Bielak et al., 2010; Stawski et al., 2019), they are related to fluctuations along longer time scales (hours, days, years) rather than the moment-to-moment variability we focus on for the purposes of this study. Future work might clarify whether within task RT variability that we examined here produces similar findings to measures of variability examined along longer time scales for choice and simple RT data (e.g., in ecological momentary assessment designs).

Here, we sought to compare and contrast different measures of cognitive performance and variability in a large, diverse sample to better characterize patterns of age-related change, establish updated norms, and compare across standard performance metrics in a large, well-powered sample. Specifically, we wanted to better understand the potential effect of aging on mean RT, RT variability (raw and mean-adjusted), and accuracy in measures of simple and choice RT. Our large sample size allowed us to look at differences in performance year-by-year across the lifespan, addressing sample size limitations of prior studies, and allowing us to estimate potential trajectories of cognitive change for different indices. We also compared different approaches to capturing RT variability to see whether they produced similar or discrepant results (Hultsch et al., 2002; Stuss et al., 2003; Dykiert et al., 2012).

We hypothesized that older age would be associated with increased mean RT and RT variability, for both raw RT variability metrics and mean-adjusted RT variability metrics, consistent with the literature described above. Like Fortenbaugh et al. (2015), who used a similar type of sample, we expected to find a non-linear relationship between age and variability in RT, distinct from the relationship of mean RT with age. We were particularly interested in the age and slope of increases in RT variability in normal aging. Our study expands on the prior literature, as it is the largest evaluation of RT variability in measures of choice (Choice RT) and simple RT (Simple RT) across the lifespan to date.

## Materials and Methods

### Participants

Participants were 12,327 visitors to TestMyBrain.org, our citizen science research platform where participants take part in research experiments to contribute to science and learn more about themselves through immediate and personalized return of research results. The protocol was approved by the Harvard Committee on the Use of Human Subjects. All data are completely deidentified and all participants provided consent. Participants were given feedback about their performance relative to other individuals who had completed the same task. Data were obtained from March 2017 to February 2018.

Participants’ ages ranged from 10 to 96 years old; the average age was 27.36 (*SD* = 13.98). After binning ages for visualization, we excluded ages that had fewer than 25 participants, which restricted our age range from 10 to 70. The sample was predominantly male (55%; female = 44%; unknown = 1%). The majority of participants were from the United States (33%) and other English speaking countries (21% from the United Kingdom, Australia, Canada, and Ireland). The highest percentage of participants identified as of European decent (49.95%), followed by Asian decent (13.97%). A plurality of our sample completed high school (*n* = 2693; 22.70%), with the next largest groups completing some college (*n* = 2094; 19.33%), college (*n* = 1982; 16.70%), and graduate school (*n* = 1745; 14.70%).

### Measures

#### Simple Reaction Time

Participants were asked to press the space bar or touch the screen whenever a red WAIT sign changed to green GO!. Participants completed three practice trials before 30 task trials. The task takes approximately 1.5 min and estimates basic psychomotor response speed with high reliability (split-half reliability based on mean RT: 0.93). Participants had 2000 ms to respond on each trial, with a variable inter-trial interval of 700–1500 ms between trials. The task was designed to capture basic psychomotor speed. For each participant, we calculated mean RT, median RT, standard deviation RT, intraindividual coefficient of variability (ICV; standard deviation in RT/mean RT), as well as mean residualized standard deviation in RT (residualized SD RT).

For both tasks, we calculated mean RT and median RT. We also calculated standard deviation RT, coefficient of variability (ICV; standard deviation in RT/mean RT), and mean residualized SD RT (Hultsch et al., 2002; Stuss et al., 2003; Dykiert et al., 2012). The first measure (standard deviation RT; hereafter, SD RT) provides a raw measure of RT variability. The second two measures provide mean-adjusted measures of RT variability, based on the observation that RT variability tends to be associated with mean RT. For the intraindividual coefficient of variation (ICV), the standard deviation is scaled by the mean (SD RT/mean RT). We also calculated a residualized SD RT, using linear regression to remove variance explained by mean RT to obtain a mean residualized standard deviation (residualized SD RT).

#### Choice Reaction Time

Participants were asked to indicate the direction of an arrow that is a different color from the rest, see Supplementary Material. The task takes approximately 2.5 min, and produces highly reliable scores (split-half reliability based on median RT: 0.81). Participants had 5000 ms to respond on each trial, with a variability inter-trial interval of 700–1500 ms between trials. This paradigm was adapted from Maljkovic and Nakayama (1994). Participants completed four practice trials before beginning the scored portion of the experiment, 30 trials. They are instructed to respond as quickly and as accurately as possible. This task was designed to capture domains of psychomotor response speed, response selection, and cognitive inhibition (due to interference effects between trials).

**FIGURE 1:**
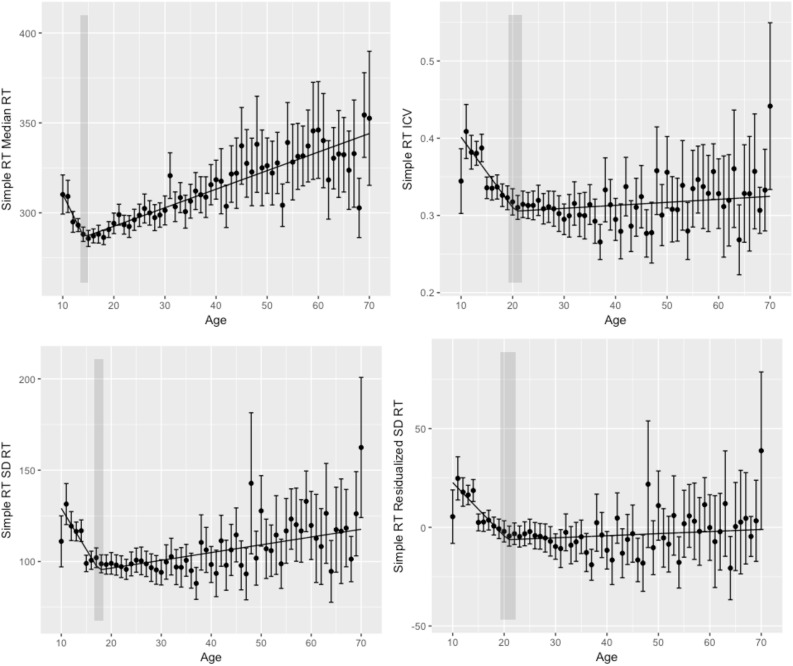
Simple reaction time performance. **(Upper left)** Simple reaction time (simple RT) median reaction time (RT) by age. **(Upper right)** Simple RT intraindivididual coefficient of variability (ICV) by age. **(Bottom left)** Simple RT standard deviation reaction time (SD RT) by age. **(Bottom right)** Simple RT residualized SD RT by age. Shaded regions indicate the 95% confidence interval around the breakpoints.

**FIGURE 2:**
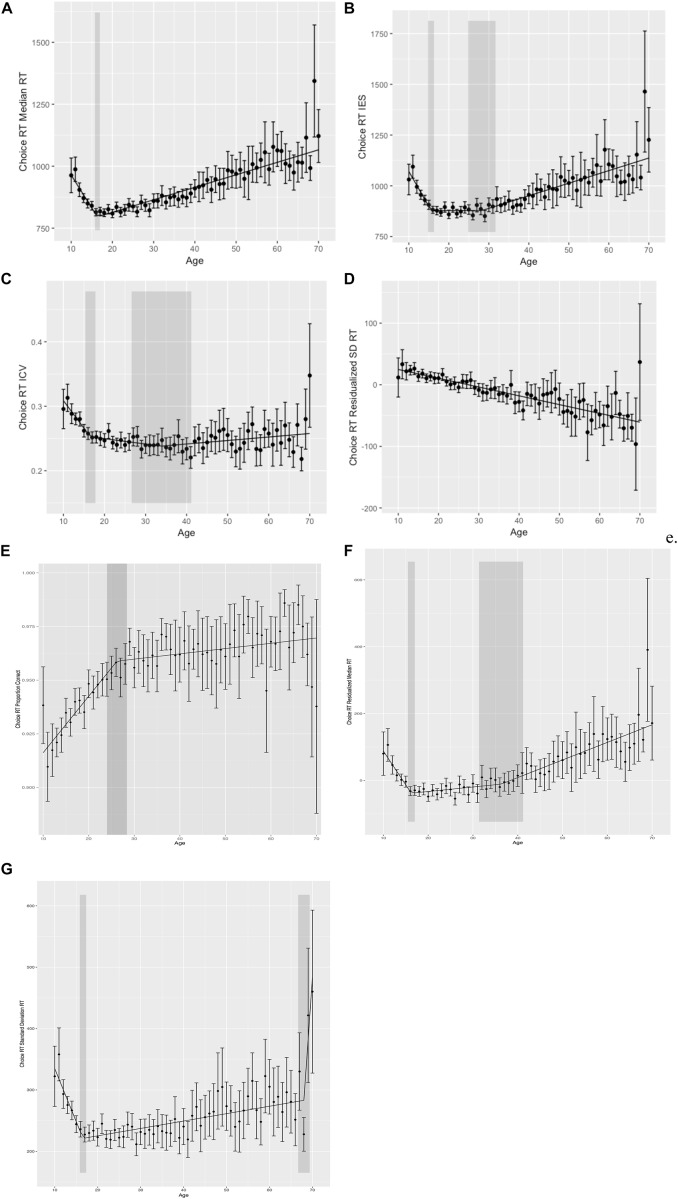
Choice reaction time performance. **(A)** Choice reaction time (choice RT) median reaction time (RT) by age. **(B)** Choice RT inverse efficiency score (IES) by age. **(C)** Choice RT intraindividual coefficient of variability (ICV) by age. **(D)** Choice RT residualized standard deviation reaction time (SD RT) by age. **(E)** Choice RT proportion corrected by age. **(F)** Choice RT residualized median RT by age. **(G)** Choice RT raw standard deviation RT by age. Shaded regions indicate the 95% confidence interval around the breakpoints.

For the choice RT, we also calculated accuracy (proportion correct) and the inverse efficiency score (IES). IES provides an accuracy-adjusted measure of response speed to account for speed accuracy trade-offs (mean RT/accuracy) (Bruyer and Brysbaert, 2011; Heitz, 2014). Finally, we looked at median RT for choice RT controlling for differences in median RT for simple RT (residualized median RT), to look at effects of age on the response selection component of choice RT after removing variance better explained by basic psychomotor response speed. Table 1 shows summary scores for measures calculated. Scores are binned by year of age in Figures 1, 2 for visualization purposes only.

**TABLE 1:** Descriptive statistics and sex differences for all performance indicators.

**Measure**	**Simple reaction time**		**Choice reaction time**	
	**Overall mean (SD)**	**Female vs. male Cohen’s *D* [95% CI]**	**Overall mean (SD)**	**Female–male Cohen’s *D* [95% CI]**
Mean RT	318 (64)	0.32 [0.28, 0.36]	919 (250)	0.24 [0.2,0.28]
Median RT	301 (60)	0.29 [0.25, 0.33]	869 (222)	0.24 [0.2,0.28]
Standard deviation RT	104 (61)	0.1 [0.06, 0.14]	246 (162)	0.19 [0.15,0.23]
Coefficient of variability (ICV)	0.33 (0.17)	−*0.004* [*−0.04*, *0.04*]	0.25 (0.11)	0.14 [0.10, 0.18]
Residualized SD RT	0 (57)	−*0.011* [−*0.05*, *0.03*]	0 (95)	*−0.015* [−*0.05*, *0.02*]
Proportion correct	NA	NA	0.95 (0.07)	0.12 [0.09, 0.17]
Inverse efficiency score (IES)	NA	NA	979 (305)	0.17 [0.13, 0.21]
Residualized median RT	NA	NA	0 (201)	0.13 [0.91, 0.17]

*Reaction times are in milliseconds. SD, standard deviation; RT, reaction time. Italicized values give non-significant sex differences (all other ps < 0.0001). Sex differences are shown for females compared with males (larger, positive values: females > males). Coefficient of variability scores were calculated as SD RT/mean RT. Inverse efficiency scores were calculated as mean RT/proportion correct. Residualized SD RT scores were calculated by calculating residuals for SD RT after controlling for mean RT. Residualized median RT scores were calculated by calculating residuals for median RT on the choice RT after controlling for median RT on the simple RT.*

### Exclusions

We first excluded participants based on data quality. Choice RT trials with very short response times (RT < 500 ms) were trimmed, based on the finding that accuracy falls to chance for trials <500 ms (see Supplementary Material). Simple RT trials with very short response times (RT < 200 ms) were trimmed based on theoretical minimum response times for visual simple RT tasks (Thorpe et al., 1996). Our sample was reduced to 10,499 after excluding people who didn’t complete both simple RT and choice RT. From there, we excluded 439 participants with more than six trimmed trials on each task (out of 30) or with chance performance or below on the choice RT (50% or below) as indicators of careless or inattentive responders. Thus, our final analytic sample size was 10,060.

### Data Analyses

Data were analyzed in RStudio Team (2016). Effect sizes are reported with 95% CIs. Given our interest in capturing cross-sectional lifespan changes in performance and performance variability, we performed segmented (piecewise) regression, a method using multiple linear segments to model non-linear changes (Muggeo, 2003, 2008) and implemented by others investigating lifespan changes in large samples (see Yeatman et al., 2014; Fortenbaugh et al., 2015). Within segmented regression analyses, the point at which the effect of one variable on another changes (breakpoint) is determined by a significant change in slope magnitude and/or direction. We provide breakpoints and discuss the ages at which the relationship between age and coefficient of variability changes. We also compared model fit of the segmented models using the Bayesian Information Criterion (BIC) and Akaike Information Criterion (AIC) to select the best model fit.

## Results

### Simple Reaction Time

Table 1 gives results of descriptive statistics for all measures of performance (median RT) and performance variability (SD RT, ICV, residualized SD RT). Men were faster and had lower standard deviations in RT than women (*p* < 0.0001). Neither ICV nor residualized SD RT, however, differed between men and women (Table 1).

To evaluate cross-sectional changes in performance across the lifespan, we performed segmented regression analyses with age as our independent variable and (1) median RT, (2) SD RT, (3) ICV, and (4) residualized SD RT as dependent variables. We chose median RT to minimize the effects of outliers. Results of segmented regression are given in Table 2 and Figure 1.

**TABLE 2:** Results of segmented regression.

**Measure**	**Best fit model**	**Age breakpoint #1 [95% CI]**	**Age breakpoint #2 [95% CI]**	Slope (b): Age 10 to breakpoint #1* **[95% CI]**	Slope (b): Breakpoint #1 to breakpoint #2* **[95% CI]**	**Slope (b): Breakpoint #2 to Age 70 [95% CI]**
**Simple reaction time**						
Median RT	Two segment	14.1 [13.4, 14.9]	NA	−5.7 [−8.5, −2.8]	1 [0.94, 1.1]	NA
Standard deviation RT	Two segment	17.8 [16.7, 18.9]	NA	−4.2 [−5.5, −3]	0.44 [0.33, 0.54]	NA
Coefficient of variability (ICV)	Two segment	20.1 [19.2, 21.2]	NA	−0.0091 [−0.011, −0.007]	0.0039 [0.00005, 0.00072]	NA
Residualized SD RT	Two segment	20.7 [19.2, 22.3]	NA	−2.7 [−3.54, −2]	0.11 [−0.004, 0.22]	NA
**Choice reaction time**						
Median RT	Two segment	17.2 [16.6, 17.8]	NA	−21.1 [−9.7, −25.3]	5.2 [4.8, 5.5]	
Standard deviation RT	Three segment	16.5 [15.9, 17.1]	68 [66.7, 69.4]	−17.3 [−21.3, −13.2]	1.2 [0.93, 1.5]	
Coefficient of variability (ICV)	Three segment	16.5 [15.3, 17.7]	35.7 [28.3, 43.1]	−0.008 [−0.01, −0.006]	−0.0008 [−0.0014, −0.0002]	0.0006 [0.00017, 0.0011]
Residualized SD RT	One segment	NA	NA	−0.0008 [−0.00094, −0.00069]	NA	NA
Proportion correct	Two segment	26.2 [24, 28.4]	NA	0.0026 [0.0022, 0.0031]	0.00025 [0.00006, 0.00043]	NA
Inverse efficiency score (IES)	Three segment	15.8 [14.9, 16.6]	28.8 [25.1, 32.5]	−33 [−42.2, −23.8]	0.28 [−1.7, 2.3]	6.2 [5.5, 7]
Residualized median RT	Three segment	16.2 [15.5, 17]	36.3 [31.4, 41.2]	−20.2 [−25.2, −15.2]	1.35 [0.4, 2.3]	5.3 [4.4, 6.1]

*Slope to next breakpoint or age 70 years, whichever occurs earlier. A four segment model was never selected based on model fit parameters. For all models, addition of a segment did not improve model fit. For model fit parameters of selected models, see Supplementary Material.

We first examined the relationship between age and median RT, with the goal of replicating previous findings of improvements in speed-related aspects of performance in adolescence, followed by declines through most or all of adulthood. Segmented regression analyses demonstrated that the relationship between age and median RT was best fit by a two segment (one breakpoint) linear function with a breakpoint at 14 years (age of best performance). Median RT decreased before 14 years and increased thereafter. These findings are consistent with the notion that processing speed declines with age (e.g., Salthouse, 1996).

Age-related differences in measures of performance variability for simple RT were largely convergent. Segmented regression analyses for SD RT, ICV, and residualized SD RT all were best fit by a two segment model, with reductions in variability from ages 10 to ages 18–21 years, and performance variability increasing thereafter for the remainder of the lifespan.

### Choice Reaction Time

Table 1 provides descriptive statistics for all measures of performance (median RT, proportion correct, IES, residualized median RT) and performance variability (SD RT, ICV, residualized SD RT). Men again were faster and had lower variability in their RTs than women (*p* < 0.0001). Women, on the other hand, were more accurate (*p* < 0.0001). There were no significant differences between men and women in residualized SD RT (*p* = 0.42) (Table 1).

To evaluate cross-sectional changes in performance across the lifespan, we again performed segmented regression analyses with age as our independent variable and (1) median RT, (2) SD RT, (3) ICV, (4) residualized SD RT, (5) proportion correct, (6) IES, and (7) residualized median RT as dependent variables. We again chose median RT to minimize the effects of outliers. Results of segmented regression for choice RT data are also given in Table 2 and Figure 2.

For median RT, segmented regression analyses again demonstrated that the relationship between age and median RT was best fit by a two segment (one breakpoint) linear function with a breakpoint at 17 years (age of best performance). Median RT decreased before 17 years and increased thereafter. Proportion correct was also best fit by a two segment (one breakpoint) model, with a breakpoint at 26 years, and improvements in performance across the entire lifespan.

The opposite and opposing relationship between response speed and accuracy indicates that speed–accuracy trade-offs play a large role in lifespan-related differences in performance and, individually, may not appropriately capture cognitive control abilities. To account for speed–accuracy tradeoffs, we looked at the relationship between age and IES (accuracy corrected RT: mean RT/proportion correct). When looking at IES, a three segment model provided best fit, with breakpoints at 16 and 29 years. IES improved from ages 10 to 16 years, increased slightly from ages 16 to 29 years, and then increased more sharply thereafter.

Median RT for choice RT controlling for median RT on simple reaction gave results that were more similar to IES than to median RT. A two breakpoint model best fit this data, with breakpoints at 16 and 36, with improvements in residualized median RT from ages 10 to 16 years, increases from 16 to 36 years, and then sharper increases from 36 to 70 years.

Age-related differences in measures of performance variability for choice RT did not converge across measures. Both SD RT and ICV across the lifespan were best fit by a three segment function, with a first breakpoint at age 16 years. For SD RT, performance variability decreased from 10 to 16, and then increased thereafter. Segmented regression identified a second breakpoint for SD RT at age 68 years, where variability increased very steeply after age 68 years. ICV, on the other hand, decreased from 10 to 16, and then continued to decrease until a second breakpoint at age 36 years, before increasing over the remainder of the lifespan. Results for residualized SD RT were markedly different, however, with a *linear decrease* across the lifespan (i.e., decreasing variability with increasing age) and no evidence of breakpoints as identified by segmented regression. In other words, there was no point at which variability increased based on analyses of residualized SD RT from choice RT data.

### Age × Sex Interactions

Men and women differed significantly across most measures of performance and performance variability, although with small effect sizes (Table 1). We further examined the interaction of Age × Sex by comparing segmented models with and without separate model lines for males and females. Results revealed no significant difference in segmented models, for any measures, indicating that the Age × Sex interactions were not significant (*p*s all > 0.2).

## Discussion

This is the largest study to compare and contrast different indices of performance and performance variability in simple RT and choice RT tasks. Different measures of performance capture different characteristics of human behavior, and here we found that different measures, including measures that putatively measure the same constructs, exhibited different patterns of age-related performance. For example, we found that the peak age for cognitive performance based on median RT was 17, whereas based on coefficient of variability scores for choice RT, we found highest performance at age 36 years. Interestingly, different measures of RT variability also showed distinct patterns across age. While measures of raw variability (SD RT) and coefficient of variability (SD RT/mean RT) showed similar differences with age, for choice RT mean residualized variability (SD RT controlling for mean RT) showed a distinct relationship with age – with apparent decreases in variability across the lifespan, contrary to the literature. This contrasted with simple RT where mean-residualized variability produced similar (but attenuated) age effects when compared with other variability measures.

Our large sample size allowed us to finely characterize variations in RT and RT variability across the lifespan. Such results can provide normative models for cognitive performance on such tasks across the lifespan, which could provide a basis for revealing abnormal trajectories (Fortenbaugh et al., 2015). Here, we were able to distinguish between different measures of cognitive performance and variability across the lifespan, demonstrating that different measures capture different aspects of cognitive performance with respect to age. For instance, our data clearly demonstrated the classic speed accuracy trade-offs that emerge across the lifespan (Bruyer and Brysbaert, 2011; Heitz, 2014), with increasing median RT (even after adjusting for basic psychomotor speed) as well as increasing accuracy on our choice RT. IES, which adjusts for speed accuracy trade-offs, showed minimal change during early adulthood, with poorer performance not emerging until age 28 years. Finally, RT variability seemed to increase around middle age for raw variability, and mean adjusted variability (coefficient of variability), although these effects were eliminated and *reversed* after controlling for mean RT.

Our findings of different ages of peak performance based on mean RT and RT variability suggest that different measures of variability might yield different information about lifespan-related processes. What is most striking about our data here was how remarkably consistent the linear changes in residualized variability were across the lifespan for choice RT, with an almost entirely linear decrease across age that could not be explained by differences in accuracy. We are not certain why the unusual residualized SD RT result appears only in the choice RT task but not simple RT; however, we suspect that it is due to the fact that responses in the choice RT reflect a more complex cognitive process than simple RT that contributes to increases in the mean with age but not SD. Although mean residualized standard deviations have become the primary method of quantifying RT variability, it may be that adjusting for the mean can sometimes obscure true differences in variability. If mean RTs increase with older age due to multiple additive processes, not all of which are associated with changes in variability of RTs, then removing variance associated with the mean could result in an overcorrection that might explain the results observed here. For example, a tendency to respond less impulsively with age would tend to offset greater variability in RTs in a way that is due to shifts in strategy that increase response time without concomitant increases in RT variability. The fact that our finding contrasts sharply with the literature may be due to task differences, file drawer effects (due to violation of *a priori* expectations), or unmeasured confounds. Our findings of reduced mean residualized variability with age should therefore be interpreted with caution until it has been replicated in another sample.

We found a small effect for differences in variability (based on raw variability and coefficient of variability) between men and women, with men demonstrating slightly more consistent performance. We cannot assume that the differences are due to sex differences in processing speed alone [men are typically faster than women, see Roivainen (2011) for a review], as variability within an individual takes average processing speed into account. Some researchers have suggested that differences in cognitive performance processes are related to differences in symptom trajectories between men and women, namely the idea that women are more vulnerable to depression while men are more vulnerable to impulse control disorders (Li et al., 2009) that may be related to differences in variability and speed–accuracy trade-offs.

There are several limitations of the current study. First, our cross-sectional design unfortunately prohibits us from making any strong conclusions related to individual lifespan trajectories as findings could be due to cohort effects or ascertainment biases that vary by age. Interpretation of correlations (slopes in segmented regression) is problematic with such unequal age bins, and thus, we emphasize patterns of change in terms of changes in slope (breakpoints) rather than correlations as point estimates. Second, given that our sample was self-selected, it is possible that our results are biased toward higher functioning older adults with more expertise using computers. The expectation is that this would cause us to *underestimate* performance decrements with age and potentially *overestimate* any performance improvements (e.g., increased accuracy). Additionally, selection biases in older adults due to mental and physical fitness has been well-documented (Ganguli et al., 1998; Golomb et al., 2012). On the other hand, our self-selected sample allows for a larger outreach to individuals from communities that are typically harder to reach including working adults, people in rural areas, or those with limited mobility or resources to participate in research studies. Third, while prior TestMyBrain.org sample studies have been replicated compared to traditionally collected and nationally representative US samples (Germine et al., 2011; Hartshorne and Germine, 2015), further validation work is recommended. Finally, our very brief measures of simple and choice RT were sufficient to produce reliable measures of psychomotor response speed, but did not allow us to employ more sophisticated model fitting techniques to trial-by-trial data that have been used in other studies of RT variability, such as estimation of multiple components of variability using an ex-Gaussian distribution (see McAuley et al., 2006; Matzke and Wagenmakers, 2009) or estimation of variability over longer time intervals, such as in a measurement burst design (Stawski et al., 2019).

Despite these limitations, our study provides a potential foundation for future research on lifespan performance and performance variability, how best to conceptualize variability, as well as a richer characterization of how performance metrics differ even in relatively simple task designs. Findings from web-based samples such as this one have been shown to match traditional findings from the literature, and are being used more often to recruit larger, more diverse samples (i.e., Nosek et al., 2002; Soto et al., 2011; Fortenbaugh et al., 2015; Hartshorne and Germine, 2015). Additionally, our pattern of mean RT across the lifespan, which peaks in the early 20s, replicates gold standard longitudinal work (e.g., Deary and Der, 2005), as we have previously shown that lifespan patterns of change in processing speed replicate work from these gold standard studies (Hartshorne and Germine, 2015). Given the utility and ease of web-based cognitive tests [see Koo and Vizer (2019) for a recent review], an approach that integrates multiple metrics may be useful for clinicians and researchers to study cognitive performance and performance variation at many stages across the lifespan.

## Author’s Note

The primary results reported in this manuscript have been presented at local and national conferences between 2018 and 2019. The data are deposited to the Open Science Framework, which can be accessed with the following link: https://osf.io/w5nge/.

## Data Availability Statement

All datasets generated for this study are included in the article/Supplementary Material. The data is available through OSF: https://osf.io/w5nge/.

## Ethics Statement

The studies involving human participants were reviewed and approved by Harvard University.

## Author Contributions

LR and LG contributed to the conception and design of the study, organized the database, and performed the statistical analysis. LR wrote the first draft of the manuscript. All authors contributed to the manuscript revision, and read and approved the submitted version of the manuscript.

## Conflict of Interest

The authors declare that the research was conducted in the absence of any commercial or financial relationships that could be construed as a potential conflict of interest.
