# Supplementary material for: Heterogeneous Indicators of Cognitive Performance and Performance Variability Across the Lifespan
Source: Front Aging Neurosci. 2020 Mar 6;12:62. doi: 10.3389/fnagi.2020.00062 (PMC7068851; doi:10.3389/fnagi.2020.00062)
Supplement: Supplementary file 1 [file Data_Sheet_1.PDF]

SUPPLEMENTAL MATERIALS

Choice Reaction Time Instructions

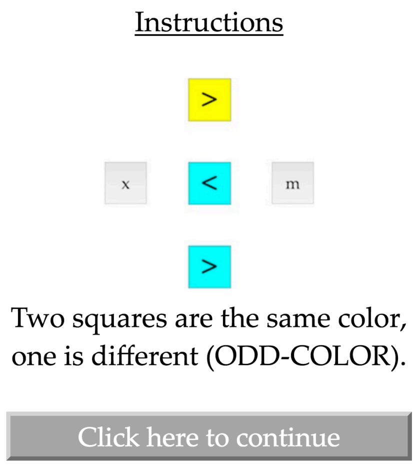

a.

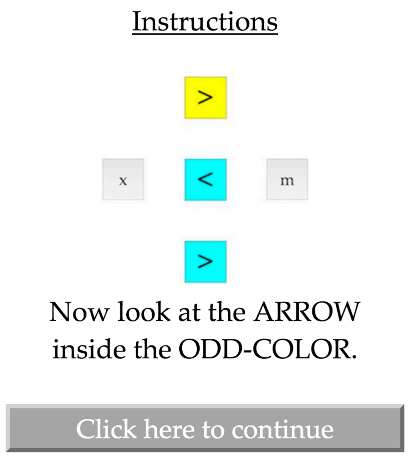

b.

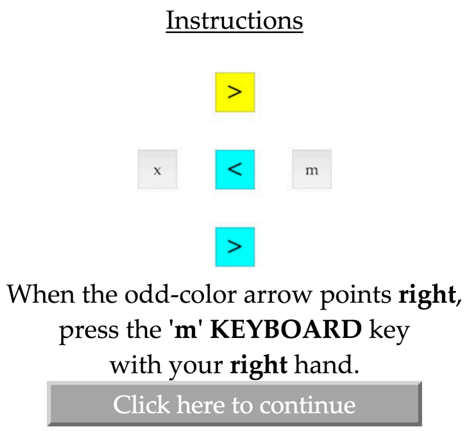

c.

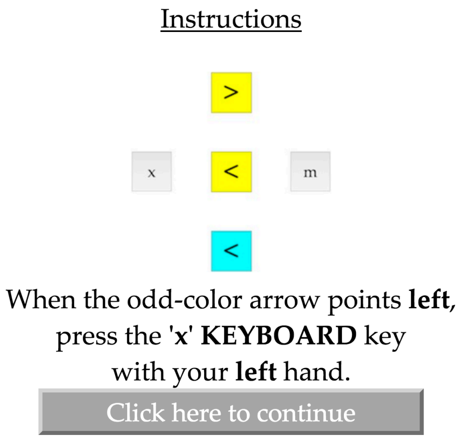

d.

Note. Instructions for Choice Reaction Time are labeled in sequential letter order, a to d. Participants complete 4 practice trials before beginning the 30 task trials.

Model Fit Parameters for Selected and Unselected Segmented Regression Models

CHOICE REACTION TIME

Median Reaction Time
LINEAR
R^2=.08, F(1,10058)=587.6, p < .0001
TWO SEGMENT
R^2=.08, F(2,10056)=141.5, p < .0001

Proportion Correct
LINEAR
R^2=.03, F(1,10058)=323, p < .0001
TWO SEGMENT
R^2=.04, F(2,10056)=60, p < .0001

Intraindividual Coefficient of Variability (ICV)
LINEAR
R^2=.004, F(1,10058)=40.3, p < .0001
TWO SEGMENT
R^2=.016, F(2,10056)=64.5, p < .0001
THREE SEGMENT
R^2=.017, F(3,10054)=6.9, p < .001

Standard Deviation RT
LINEAR
R^2=.002, F(1,10058)=20.9, p < .0001
TWO SEGMENT
R^2=.022, F(2,10056)=103.6, p < .0001
THREE SEGMENT
R^2=.026, F(3,10054)=21.9, p < .0001

Inverse Efficiency Score
LINEAR
R^2=.025, F(1,10058)=264, p < .0001
TWO SEGMENT
R^2=.054, F(2,10056)=150.9, p < .0001
THREE SEGMENT

R^2=.057, F(2,10054)=16.2, p < .0001

Residualized Standard Deviation
LINEAR
R^2=.044, F(1,10058)=463, p < .0001

Residualized Median Reaction Time
LINEAR
R^2=.024, F(1,10058)=251.2, p < .0001
TWO SEGMENT
R^2=.048, F(2,10056)=124.9, p < .0001
THREE SEGMENT
R^2=.05, F(2,10054)=15.6, p < .0001

SIMPLE REACTION TIME

Median Reaction Time
LINEAR
R^2=.048, F(1,10058)=511.4, p < .0001
TWO SEGMENT
R^2=.052, F(2,10056)=22, p < .0001

Standard Deviation Reaction Time
LINEAR
R^2=.0007, F(1,10058)=8.0, p < .01
TWO SEGMENT
R^2=.014, F(2,10056)=69.9, p < .0001

Coefficient of Variability (ICV)
LINEAR
R^2=.0043, F(1,10058)=44.2, p < .0001
TWO SEGMENT
R^2=.019, F(2,10056)=76.1, p < .0001

Residualized Standard Deviation
LINEAR
R^2=.004, F(1,10058)=42.2, p < .0001
TWO SEGMENT
R^2=.016, F(2,10056)=63.5, p < .0001

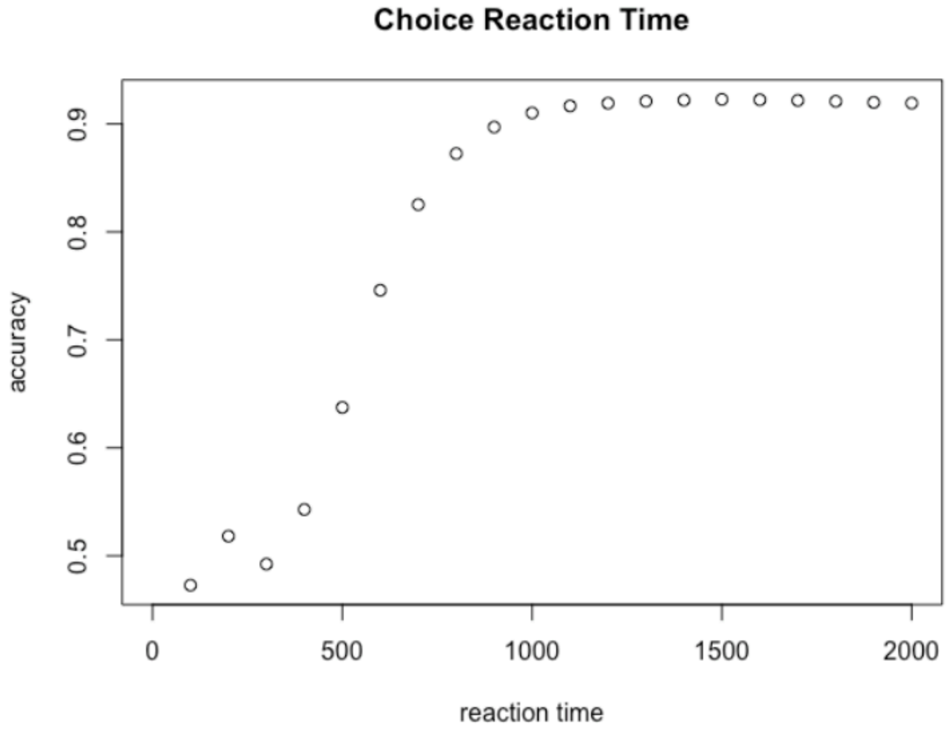

Figure determines minimum reaction time cut offs for trimming trials (< 500ms)
